# Acute eosinophilic pneumonia with detection of pure *Tropheryma whipplei* in bronchoalveolar lavage fluid: a case report

**DOI:** 10.3389/fmed.2025.1683834

**Published:** 2025-10-03

**Authors:** Yi Hu, Jie Zhang, Ting Wang, Bing Xue

**Affiliations:** Department of Respiratory and Critical Care Medicine, Chuiyangliu Hospital Affiliated with Tsinghua University, Beijing, China

**Keywords:** acute eosinophilic pneumonia, *Tropheryma whipplei*, targeted next-generation sequencing, cough, bronchoalveolar lavage fluid

## Abstract

Acute eosinophilic pneumonia (AEP) is a rare but potentially severe respiratory illness. *Tropheryma whipplei* can also cause acute pulmonary infection, leading to atypical symptoms and computerized tomography (CT) findings. We report a case of acute eosinophilic pneumonia that was misdiagnosed as a *Tropheryma whipplei* infection based on targeted next-generation sequencing (tNGS) of the bronchoalveolar lavage (BAL) fluid. A 51-year-old male firefighter was admitted with a 7-day history of dry cough and 1 day of fever, initially diagnosed with community-acquired pneumonia and treated empirically with antibiotics. TNGS of BAL fluid identified only *Tropheryma whipplei*, leading to a misdiagnosis of *T. whipplei* pneumonia. Despite antibiotic treatment, the patient’s condition worsened, and a revised diagnosis of AEP was made based on persistent eosinophilia and pathological findings. Treatment with methylprednisolone led to rapid improvement. The detection of a high sequence count of *Tropheryma whipplei* as the sole bacterium in patients suspected of having acute eosinophilic pneumonia can hinder the diagnosis and treatment of the patient, highlighting the importance of differential diagnosis and the order of treatment choices.

## Introduction

Eosinophilic pneumonia (EP) is a rare and diverse group of pulmonary conditions characterized by the presence of eosinophils in the alveolar spaces, interstitium, or peripheral blood. It can be classified into acute and chronic forms, with acute eosinophilic pneumonia (AEP) typically presenting as a rapid onset of fever, cough, dyspnea, and diffuse pulmonary infiltrates on imaging studies (1, 2). The etiology of AEP is often idiopathic but can be associated with smoking, inhalational exposures, medications, and infections (1, 3, 4). Diagnosis relies on the demonstration of pulmonary eosinophilia in bronchoalveolar lavage (BAL) fluid and exclusion of other causes (1). *Tropheryma whipplei* is associated with chronic multisystem infections, including gastrointestinal involvement, but can also cause acute pulmonary infections (5, 6). The clinical symptoms of *Tropheryma whipplei* pneumonia are nonspecific, often leading to misdiagnosis or underdiagnosis (7). The advent of next-generation sequencing (NGS) has significantly enhanced the ability to diagnose rare pathogens like *Tropheryma whipplei*, providing a solid foundation for precise and effective treatment (8).

In this case report, we present a patient who was initially misdiagnosed with *Tropheryma whipplei* pneumonia due to the nonspecific clinical presentation and the use of targeted NGS (tNGS), which ultimately revealed a diagnosis of eosinophilic pneumonia. This case highlights the importance of considering a broad differential diagnosis and the potential pitfalls of relying solely on advanced molecular techniques without a thorough clinical correlation.

## Case report

The patient is a 51-year-old male firefighter who was admitted to the hospital due to “dry cough for 7 days and fever for 1 day.” The highest recorded body temperature was 38.0 °C. He has a 6-year history of hypertension, with the highest blood pressure reaching 190/100 mmHg. He was previously taking allisartan isoproxil but discontinued it 6 months ago due to hypotension. Six years ago, he was diagnosed with coronary heart disease and had a stent placed in the opening of the anterior descending artery; however, he has not been taking aspirin recently because of bleeding. He has no history of long-term use of immunosuppressants or hormones. The patient has a long history of smoking (40–60 cigarettes per day) and drinking. He denies any recent history of drunken aspiration, exposure to epidemic or wastewater, travel history, or medication use. The patient has poor oral hygiene and keeps a parrot.

Upon admission, the patient’s vital signs were as follows: temperature 36.0 °C, heart rate 95 beats per minute, respiratory rate 20 breaths per minute, blood pressure 94/68 mmHg, and oxygen saturation 96% (on 2 L/min of oxygen via nasal cannula). The patient appeared feckless, had diminished breath sounds on the right lung, and no dry or wet rales were heard in both lungs. There were no other obvious positive physical findings.

**Figure 1:**
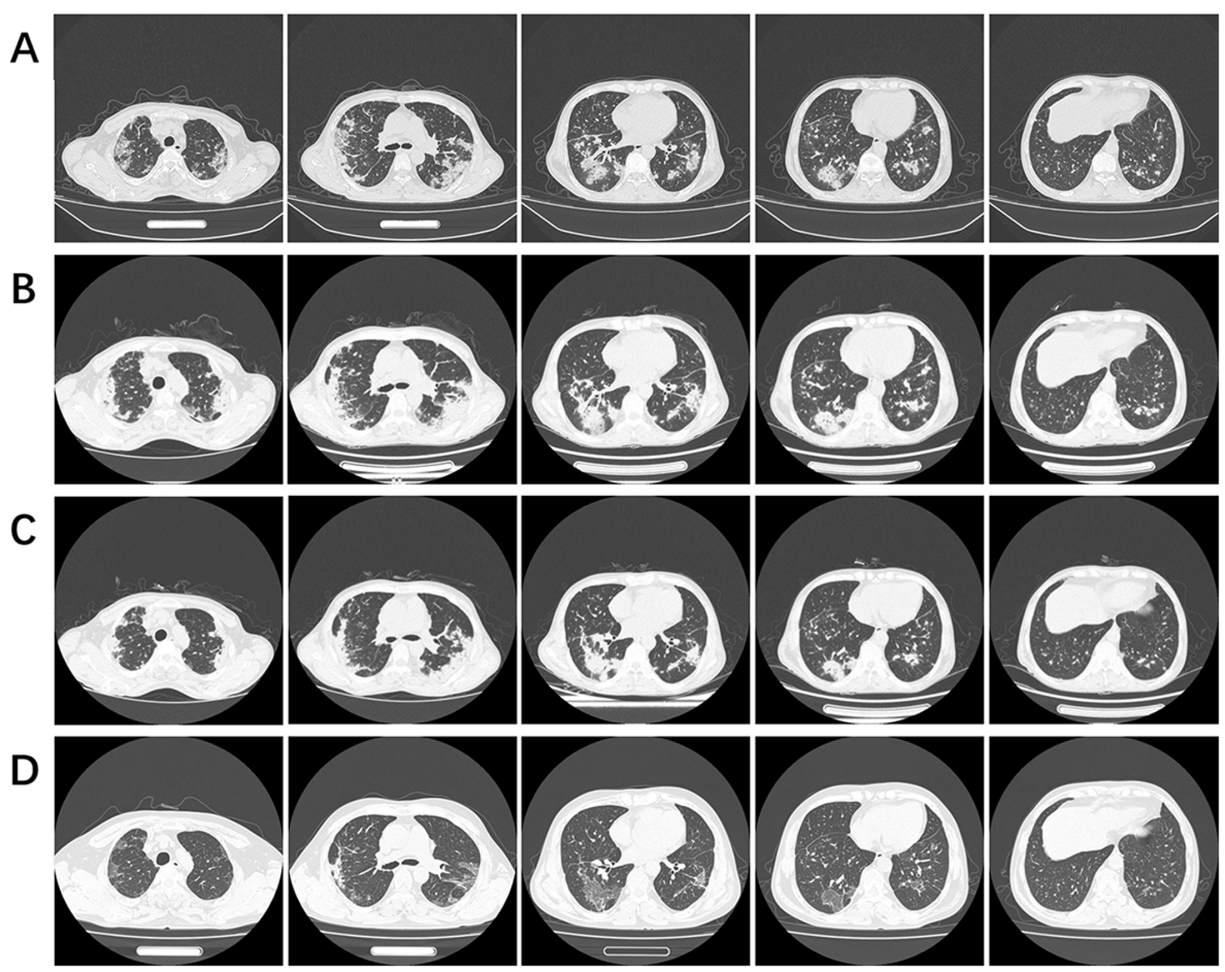
Chest CT images obtained after different treatment phases. **(A)** Upon admission; **(B)** after 7 days of treatment with meropenem (1 g q12h) and sulfamethoxazole (two tablets bid); **(C)** after 3 days of methylprednisolone (40 mg) treatment; **(D)** 2 months after sequential oral methylprednisolone treatment.

The important laboratory test results are shown in Table 1. Chest computerized tomography (CT) revealed multiple ground-glass opacities and consolidations in both lungs, mainly in the upper lobes, with air bronchograms visible in some areas (Figure 1A). Etiologically, sputum culture showed no significant bacterial growth, and nucleic acid tests for common respiratory viruses such as novel coronavirus, influenza virus, and respiratory syncytial virus were all negative.

**Table 1:** Main results of laboratory tests.

Laboratory tests	Main results
Complete blood count	White blood cell count 13.8 × 10^9^/L (reference value 3.5–9.5 × 10^9^/L), neutrophil count 9.2 × 10^9^/L (reference value 1.8–6.3 × 10^9^/L), monocyte count 0.9 × 10^9^/L (reference value 0.1–0.6 × 10^9^/L), eosinophil count and 2.46 × 10^9^/L (reference value 0.02–0.52 × 10^9^/L)
Inflammatory markers	C-reactive protein 64.32 mg/L (reference value 0–5 mg/L), procalcitonin 0.36 ng/mL, erythrocyte sedimentation rate 83 mm/h (reference value 0–15 mm/h)
Blood gas analysis	pH 7.443, partial pressure of oxygen 58.9 mmHg, partial pressure of carbon dioxide 37.1 mmHg, oxygen saturation 90.5%, lactate 2.11 mmol/L, actual bicarbonate 24.8 mmol/L, and standard bicarbonate 25.1 mmol/L
Biochemical tests	Creatine kinase MB isoenzyme 0.1 ng/mL, alanine aminotransferase 10.0 U/L, aspartate aminotransferase 11.7 U/L, lipase 63.0 U/L, calcium 2.02 mmol/L, blood amylase 18.0 U/L, potassium 3.39 mmol/L, and sodium 129.5 mmol/L
Coagulation tests	Prothrombin time 12.3 s and fibrinogen 6.740 g/L

Based on the patient’s clinical presentation and CT findings, a preliminary diagnosis of “community-acquired pneumonia” was made, and empirical treatment with moxifloxacin was initiated. However, due to the extensive involvement of both lungs with multiple lobes affected, a bronchoscopy was performed to further clarify the diagnosis. The BAL fluid tNGS revealed *Tropheryma whipplei* as the sole identified bacterium, with a uniform sequence count of 13,665 and an estimated microbial concentration of >1.0 × 10^6^ copies/mL. Considering the low likelihood of contamination during bronchoscopic procedures and the fact that *Tropheryma whipplei* pneumonia can present with similar CT findings, the antibiotic regimen was adjusted to meropenem (1 g q12h) and sulfamethoxazole (two tablets bid). After 7 days of this antibiotic therapy, a follow-up showed an increase in blood eosinophils to 2.86 × 10^9^/L and a decrease in C-reactive protein (CRP) to 20.00 mg/L, but the chest CT revealed worsening of the pulmonary lesions (Figure 1B), indicating clinical treatment failure. Concurrently, the cellular classification of the BAL fluid showed eosinophils accounting for 40%, with no obvious bacteria, fungi, or acid-fast stained positive organisms were observed, and the pathological examination of lung tissue obtained during bronchoscopy reported: observation of bronchiolar mucosa and alveolar tissue, with alveolar tissue accounting for 60%; partial bronchiolar mucosal epithelial cell exfoliation, infiltration of eosinophils and neutrophils in the submucosa of the bronchioles, widened alveolar septa, fibrous tissue proliferation, infiltration of eosinophils and neutrophils, mild hyperplasia of alveolar epithelium, and the presence of histiocytes, eosinophils, and pink-staining homogeneous secretions within the alveolar spaces. Given the persistently high eosinophil count since admission, the patient was ultimately diagnosed with “eosinophilic pneumonia” combined with the pathological findings. Following the definitive diagnosis, the original antibiotics were discontinued, and methylprednisolone 40 mg was administered intravenously once daily. After 3 days, the patient’s symptoms improved rapidly. A follow-up complete blood count showed a decrease in eosinophils to 1.15 × 10^9^/L and a CRP level of 5.00 mg/L, with the chest CT showing some absorption of the pulmonary lesions (Figure 1C). The patient was discharged with a sequential oral methylprednisolone regimen. At the 2-month follow-up, the patient reported no recurrence of fever or cough, with significant improvement in symptoms, and the chest CT showed marked absorption of the pulmonary lesions (Figure 1D).

## Discussion

AEP is a rare but potentially severe respiratory illness that can lead to acute respiratory distress syndrome and death, with an incidence rate of approximately 9.1–11.0 per 100,000 person-years (1). While the disease can be idiopathic, known causes associated with AEP include inhalation exposures (such as tobacco, dust), the use of certain medications (such as antimicrobials), and infections (parasitic, fungal, and viral) (1). The onset of illness in this patient may be related to their occupation as a firefighter and a long history of heavy smoking. The clinical manifestations of AEP primarily include an acute onset of dry cough, dyspnea, and fever within 4 weeks, as well as less common symptoms such as malaise, myalgia, night sweats, chills, and pleuritic chest pain. These symptoms are not specific (1, 9). Currently, the modified Philit criteria are used to diagnose AEP, which include an acute respiratory illness of less than or equal to 1 month in duration, pulmonary infiltrates seen in chest X-rays or CT, more than 25% eosinophils in BAL fluid, and the absence of other pulmonary eosinophilic diseases, including eosinophilic granulomatosis with polyangiitis (Churg–Strauss syndrome), hypereosinophilic syndrome, and allergic bronchopulmonary aspergillosis (9). The main CT findings in AEP are bilateral patchy ground-glass opacities, often accompanied by consolidation and smooth interlobular septal thickening. Other common findings include thickening of the bronchovascular bundles, lymphadenopathy, poorly defined centrilobular nodules, and small to moderate bilateral pleural effusions (1).

Previous studies have reported that *Tropheryma whipplei* can be detected in 3.1–6.1% of bronchoalveolar lavage fluids (10, 11), with a detection rate of approximately 3% in the bronchoalveolar lavage fluid of patients with pneumonia (12). Increasing evidence supports the role of *Tropheryma whipplei* in acute respiratory infections, particularly when it is the sole bacterium identified (13). Among patients with *Tropheryma whipplei*-positive bronchoalveolar lavage fluid, symptoms may include not only non-specific respiratory symptoms but also abdominal pain, diarrhea, and arthralgia. CT scans may also reveal ground-glass opacities, pulmonary nodules, pleural thickening, and exudative lesions (10). Due to the atypical symptoms and CT findings of AEP and *Tropheryma whipplei* pneumonia, as well as the discovery of a high sequence count of *Tropheryma whipplei* as the sole bacterium in the bronchoalveolar lavage fluid, the initial diagnosis considered was *Tropheryma whipplei* pneumonia.

The therapeutic regimen we selected for *Tropheryma whipplei* pneumonia was meropenem (1 g q12h) combined with sulfonamides (two tablets bid), which has been reported to be effective in previous cases (14, 15). However, after 7 days of antimicrobial therapy, the patient’s symptoms did not improve, and pulmonary imaging showed further deterioration. Considering the pathological results, the diagnosis was revised to “acute eosinophilic pneumonia.” Consequently, *Tropheryma whipplei* was considered to be colonizing rather than causing disease. This underscores that molecular findings can only be reliably interpreted as indicative of a true pathogen and guide targeted therapy when demonstrating high concordance with the clinical–imaging–microbiological–pathological evidence chain, particularly in complex or diagnostically challenging cases. Otherwise, they should be regarded as likely representing colonization or contamination to prevent misdirection of clinical management. Subsequently, the treatment was adjusted to methylprednisolone 40 mg qd administered intravenously. Improvement in symptoms, laboratory findings, and pulmonary imaging was observed just 3 days after the initiation of this treatment. Due to the severity of the initial pulmonary lesions, we extended the duration of corticosteroid therapy. Over time, the patient’s symptoms gradually resolved, and pulmonary imaging showed significant improvement. The relationship between *Tropheryma whipplei* and AEP remains unclear. However, a previous study has found a higher prevalence of *Tropheryma whipplei* infection in eosinophilic asthma (16). Additionally, there is a case report of a patient with Whipple’s disease who developed eosinophilic vasculitis (17). Future studies should include expanded clinical research to substantiate the association between *Tropheryma whipplei* and eosinophilic inflammation, coupled with mechanistic investigations to validate the underlying molecular pathways involved.

## Conclusion

In conclusion, the detection of a high sequence count of *Tropheryma whipplei* as the sole bacterium in patients suspected of having acute eosinophilic pneumonia can hinder the diagnosis and treatment of the patient, highlighting the importance of differential diagnosis and the order of treatment choices. The relationship between *Tropheryma whipplei* and AEP warrants further investigation.

## Data Availability

The raw data supporting the conclusions of this article will be made available by the authors, without undue reservation.
